# Yerba Mate (*Ilex paraguariensis*) and Rheumatoid Arthritis: A Systematic Review of Mechanistic and Clinical Evidence

**DOI:** 10.3390/nu17243853

**Published:** 2025-12-10

**Authors:** Manuela Cassotta, Qingwei Cao, Haixia Hu, Carlos Rabeiro Martinez, Luis Alonso Dzul Lopez, Santos Gracia Villar, Maurizio Battino, Francesca Giampieri

**Affiliations:** 1 Research Group on Food, Nutritional Biochemistry and Health, Universidad Europea del Atlántico, Isabel Torres 21, 39011 Santander, Spain; manuela.cassotta@uneatlantico.es (M.C.); luis.dzul@unini.edu.mx (L.A.D.L.); santos.gracia@uneatlantico.es (S.G.V.);; 2 Joint Laboratory on Food Science, Nutrition, and Intelligent Processing of Foods, Polytechnic University of Marche, Italy, Universidad Europea del Atlántico Spain and Jiangsu University, China, at Universidad Europea del Atlántico, 39011 Santander, Spain; 3 Department of Clinical Sciences, Università Politecnica delle Marche, 60131 Ancona, Italy; q.cao@pm.univpm.it (Q.C.); h.hu@pm.univpm.it (H.H.); c.l.rabeiro@pm.univpm.it (C.R.M.); 4 Joint Laboratory on Food Science, Nutrition, and Intelligent Processing of Foods, Polytechnic University of Marche, Italy, Universidad Europea del Atlántico Spain and Jiangsu University, China, at the Polytechnic University of Marche, 60130 Ancona, Italy; 5 Faculty of Nutrition and Dietetics, Universidad Internacional Iberoamericana, Campeche 24560, Mexico; 6 Research Group on Food, Nutritional Biochemistry and Health, Universidad de La Romana, La Romana 22000, Dominican Republic; 7 Faculty of Human Nutrition and Dietetics, Universidade Internacional do Cuanza, Cuito EN250, Bié, Angola; 8 International Joint Research Laboratory of Intelligent Agriculture and Agri-Products Processing, Jiangsu University, Zhenjiang 212013, China

**Keywords:** yerba mate, rheumatoid arthritis, anti-inflammatory activity, antioxidant activity, bone remodeling

## Abstract

**Background:** Rheumatoid arthritis (RA) is a chronic autoimmune disease driven by persistent inflammation and oxidative stress. *Ilex paraguariensis* (yerba mate) contains bioactive compounds—particularly chlorogenic acids, quercetin, and rutin—with documented antioxidant and anti-inflammatory properties. **Objectives:** To systematically review the mechanistic and clinical evidence on *Ilex paraguariensis* and its main constituents in RA-relevant inflammatory, oxidative, and bone metabolic pathways. **Methods:** Following PRISMA 2020, PubMed/MEDLINE, LILACS, and SciELO were searched up to September 2025. Eligible studies included yerba mate preparations (last 10 years) or isolated compounds (last 5 years) assessing RA-relevant clinical, inflammatory, oxidative, or bone metabolic outcomes. Non-original studies were excluded. Owing to heterogeneity, findings were narratively synthesized, and risk of bias was evaluated using RoB 2, ROBINS-I, OHAT, and SYRCLE. **Results:** Twenty-three studies met inclusion criteria: 11 human (clinical or observational), 7 human-based in vitro, and 5 animal studies. Interventions with yerba mate infusions or standardized extracts suggest reductions in inflammatory markers (e.g., C-reactive protein, interleukin-6) and indicate improvements in glutathione-related oxidative balance. Evidence from isolated compounds, particularly quercetin and rutin, suggests comparable anti-inflammatory and antioxidant effects. Preclinical studies appear to indicate modulation of inflammatory and redox pathways relevant to RA. **Conclusions:** Yerba mate and its constituents show preliminary indications of anti-inflammatory and antioxidant effects with potential relevance to RA pathophysiology. However, in the absence of clinical trials in RA patients, conclusions remain tentative, constrained by small sample sizes, methodological heterogeneity, species differences, and internal validity concerns. Future research should include rigorously designed randomized trials and mechanistic studies using advanced human-relevant platforms, such as organoids and organ-on-chip systems.

## 1. Introduction

Rheumatoid arthritis (RA) is a chronic, systemic autoimmune disease characterized by persistent synovial inflammation, progressive joint destruction, pain, and functional disability. It affects approximately 0.5–1% of the global population, with a higher prevalence in women, and represents a major cause of impaired quality of life and work disability worldwide [1].

The pathogenesis of RA is multifactorial and involves persistent activation of innate and adaptive immune responses. Central mechanisms include the upregulation of nuclear factor kappa-B (NF-κB) and Janus kinase/signal transducer and activator of transcription (JAK/STAT) signaling, leading to increased production of pro-inflammatory cytokines such as tumor necrosis factor-α (TNF-α), interleukin (IL)-1β, IL-6, and IL-17. Fibroblast-like synoviocytes (FLSs) acquire an aggressive phenotype, contributing to pannus formation, secretion of matrix metalloproteinases (MMPs), and cartilage destruction. Oxidative stress further amplifies inflammation and tissue damage by excessive generation of reactive oxygen species (ROS) and impaired antioxidant defenses. Bone remodeling is dysregulated through activation of the receptor activator of nuclear factor-κB ligand (RANKL)/osteoprotegerin (OPG) axis, favoring osteoclastogenesis and bone erosion. Collectively, these processes sustain chronic inflammation, pain, and joint destruction [2].

Although advances in disease-modifying antirheumatic drugs (DMARDs), including biologics and Janus kinase inhibitors, have significantly improved disease control and outcomes, current therapies are not curative. Many patients experience incomplete responses, treatment-related adverse effects, or progressive structural damage despite therapy [3]. This scenario underscores the ongoing need for complementary strategies targeting key pathogenic mechanisms of RA, such as chronic inflammation, oxidative stress, FLS hyperplasia, pain, and bone remodeling.

*Ilex paraguariensis*, popularly known as yerba mate, is a species native to temperate and subtropical regions of South America, belonging to the Aquifoliaceae family. Traditionally consumed in countries such as Argentina, Brazil, Paraguay, and Uruguay, its popularity has been steadily expanding worldwide, with increasing consumption reported in Europe, North America, and parts of Asia, both as traditional infusions and as commercial preparations (e.g., energy drinks, dietary supplements, and functional foods).

Yerba mate have several therapeutic properties and are recommended as hypocholesterolemic, hepatoprotective, diuretic, digestive, nervous system stimulant, antioxidant, anti-inflammatory, antirheumatic, and lipolytic agent. It is also indicated for the treatment of occasional asthenia and as an adjunct in the treatment of overweight [4,5].

Yerba mate is particularly rich in bioactive compounds, mainly belonging to three classes: phenolic acids, methylxanthines, and flavonoids. Among phenolic acids, caffeoylquinic derivatives (chlorogenic acids) are the most abundant, accounting for nearly one third of the total polyphenolic content and including 3-caffeoylquinic (CQA), 4-CQA, 5-CQA, and dicaffeoylquinic isomers. Flavonoids, though less concentrated than phenolic acids, are consistently present, with rutin as the most abundant (up to 7–8 mg/g dry leaves), alongside quercetin and kaempferol [6]. Collectively, these compounds confer strong antioxidant and anti-inflammatory potential, which provides the biological rationale for exploring their relevance in the context of RA. A recent systematic review of clinical trials reported that dietary polyphenols, of which *Ilex paraguariensis* is a particularly rich source, may improve disease activity, reduce C-Reactive Protein (CRP) and Erythrocyte Sedimentation Rate (ESR), and modulate oxidative stress parameters in patients with RA [7]. Additional reviews have noted that dietary polyphenols may exert immunomodulatory and antioxidant effects relevant to RA pathophysiology, further supporting the interest in plant-derived phenolic compounds [8,9]. Mechanistic analyses further indicate that polyphenolic compounds can modulate key intracellular pathways involved in RA, including NF-κB, JAK/STAT, MAPKs, and Nrf2 [10].

This systematic review examines the effects of yerba mate preparations consumed in its various traditional and commercial forms and—secondarily—of its main isolated bioactive constituents on major pathogenic mechanisms of RA, including chronic inflammation, oxidative stress, FLS activation, joint damage, bone remodeling, and pain modulation.

## 2. Methods

The review was conducted in accordance with PRISMA 2020. The protocol was registered on the Open Science Framework (OSF): (https://osf.io/zd9mq, accessed on 12 November 2025).

### 2.1. Search Strategy and Information Sources

We systematically searched PubMed/MEDLINE, LILACS, and SciELO, with the last search performed on 15 September 2025. The search strategy combined terms related to *Ilex paraguariensis* or “yerba mate” and its principal bioactive constituents (e.g., “chlorogenic acid”, “caffeoylquinic acid”, “quercetin”, “ursolic acid”, “rutin”) together with “rheumatoid arthritis”. The complete search strings, including Boolean operators and MeSH terms, are provided in Appendix A. Reference lists of all included articles and related records suggested by the databases were also screened to identify additional eligible studies.

### 2.2. Inclusion Criteria

We included original studies in humans, human-derived cells, or animal models that evaluated *Ilex paraguariensis* preparations—such as traditional infusions, aqueous or hydroalcoholic extracts, or commercial formulations—or isolated bioactive constituents recognised as major components of yerba mate (including chlorogenic and caffeoylquinic acids, quercetin, and rutin). Eligible designs comprised randomized or non-randomized clinical trials, observational studies, human-based in vitro investigations, and in vivo animal studies using established models of arthritis, inflammation, or oxidative stress. Studies were required to report at least one outcome relevant to RA, including clinical parameters (e.g., DAS28, CRP, ESR), systemic inflammatory or oxidative biomarkers, or mechanistic endpoints involving RA-related pathways such as NF-κB, JAK/STAT, Nrf2 activation, fibroblast-like synoviocyte behaviour, cytokine release, or alterations in the RANKL/OPG axis. To ensure that the evidence synthesized reflects up-to-date research, we applied a ten-year window for studies on yerba mate preparations, consistent with the primary focus of the review on the preparations themselves rather than isolated constituents. For isolated bioactive compounds, a five-year window was adopted to capture mechanistic investigations generated with modern analytical approaches and more recent experimental models.

### 2.3. Exclusion Criteria

We excluded animal studies investigating isolated compounds, non-human in vitro models, non-original articles, studies lacking primary data or RA-relevant outcomes, grey literature, articles without full text available despite reasonable retrieval attempts, articles not published in English or Spanish, and any study outside the predefined time windows.

### 2.4. Grouping for Synthesis

For the narrative synthesis, studies were organised into four predefined categories: human studies assessing yerba mate infusions or non-fractioned extracts; human studies evaluating isolated bioactive constituents; human-based in vitro mechanistic studies; and in vivo animal studies using yerba mate preparations. The substantial heterogeneity in study design, populations, interventions, and outcomes did not allow for a meta-analysis, and findings were therefore synthesised qualitatively within each group.

### 2.5. Study Selection

All records were imported into Zotero, where duplicates were removed manually. Two reviewers independently screened titles and abstracts and subsequently assessed full texts against the predefined eligibility criteria. Disagreements were resolved by consensus or, when needed, through consultation with a third reviewer. No automation tools were used at any stage of the selection process. The study selection workflow is presented in the PRISMA flow diagram (Supplementary File S1).

### 2.6. Data Extraction and Quality Assessment

Two reviewers independently extracted data using a standardised form, including study design, population or model, intervention characteristics, outcomes, and main findings. Risk of bias was evaluated with design-appropriate tools: RoB 2 for randomized trials [11], ROBINS-I for observational studies [12], the OHAT tool for in vitro studies, and the SYRCLE tool for animal experiments [13].

## 3. Results

A total of 1218 records were identified through database searches and manual screening of reference lists and related articles. After removal of duplicates, 913 records were screened by title and abstract, of which 840 were excluded. Full texts of 73 articles were assessed for eligibility, leading to the inclusion of 23 studies in the final review. The study selection process is detailed in the PRISMA flow diagram (Supplementary File S1).

Of the included studies, 11 were human clinical investigations, 7 were in vitro studies using human cells, and 5 were animal models of arthritis or related inflammatory conditions. The included human studies showed marked heterogeneity in populations (healthy, cardiometabolic, HIV, postmenopausal), yerba mate formulations (infusion, roasted tea, standardized extracts, or isolated compounds), doses, exposure durations, and outcomes assessed. The main characteristics and findings of the included studies are summarized in Table 1 and Table 2.

### 3.1. Human Clinical Trials and Human-Based In Vitro Studies with Yerba Mate Infusions or Non-Fractionated Extracts

A total of ten human-based studies were identified, including seven clinical investigations and three in vitro studies evaluating *Ilex paraguariensis* (yerba mate) infusions or non-fractionated extracts (Table 1). Clinical trials conducted in metabolic, cardiovascular, HIV, or healthy populations, assessed inflammatory and oxidative stress biomarkers after short- to medium-term interventions (8 days to 4 weeks). Reported effects included reductions in circulating cytokines and inflammatory markers (e.g., CRP, IL-6, TNF-α), alongside improvements in oxidative balance (e.g., increased antioxidant capacity, improved GSH-related parameters). For instance, encapsulated dried mate extract reduced CRP and IL-6 in the higher-risk cardiometabolic subgroup [14], and roasted mate tea consumption reduced a broad panel of inflammatory cytokines [16]. In healthy men, short-term mate supplementation decreased TNF-α and IL-6 levels and increased the GSH:GSSG ratio [17,18]. One observational study reported higher bone mineral density and lower osteoporosis prevalence among habitual consumers (>1 L/day) [24]. Three in vitro studies further supported these observations. In human HepG2 hepatocytes, exposure to a yerba mate phenolic extract and its main metabolites—dihydrocaffeic acid (DHCA) and dihydroferulic acid (DHFA)—reduced intracellular ROS and lipid peroxidation, increased glutathione levels, and normalized antioxidant enzyme activities [27]. Both the native extract and DHCA were effective, whereas DHFA showed only partial activity. In endothelial cells, yerba mate extract mitigated TNF-α–induced oxidative stress and improved nitric oxide synthase (eNOS) expression (Wang et al., 2019 [30]). In THP-1 macrophages, aqueous yerba mate extract inhibited NLRP3 inflammasome activation and decreased ROS and nitric oxide levels (Santos et al., 2025 [31]).

### 3.2. Human-Based Studies with Isolated or Chemically Defined Bioactive Compounds

Nine studies investigated *Ilex paraguariensis* bioactive constituents, including chlorogenic acids, rutin, and quercetin (Table 1). Five randomized clinical trials evaluated these compounds in non-RA populations. Quercetin supplementation (500–1000 mg/day for 7 days to 12 weeks) in post-myocardial infarction patients, individuals undergoing coronary artery bypass surgery, and healthy postmenopausal women led to reductions in pro-inflammatory cytokines and increases in total antioxidant capacity, while effects on CRP varied across studies [21,22,23].

In patients with type 2 diabetes, rutin (500 mg/day for 3 months) decreased inflammatory and oxidative stress biomarkers and increased antioxidant capacity [20]. A crossover trial with a caffeoylquinic acid–standardized dry yerba mate extract also reported reductions in CRP and IL-6 in a higher cardiometabolic-risk subgroup [14].

Four in vitro studies using human-derived cells evaluated the effects of bioactive constituents present in *Ilex paraguariensis* (Table 1). Quercetin treatment of RA fibroblast-like synoviocytes (RA-FLSs) reduced the expression of the long non-coding RNA XIST, a regulator implicated in synovial fibroblast activation, and Proteasome subunit beta type-8 (PSMB8), a component of the immunoproteasome involved in antigen processing and inflammatory signaling; this downregulation was associated with reduced synoviocyte proliferation and inflammatory mediator release [28].

In RA-FLSs, quercetin also inhibited abnormal migration and invasion by upregulating microRNA-146a (miR-146a) and downregulating GATA-binding protein 6 (GATA6), leading to reduced F-actin organization; given that synoviocyte hypermigration and invasiveness drive pannus formation and joint destruction in RA, these findings support a potential anti-invasive effect [25].

In human PBMCs cocultured with mesenchymal stem cells, quercetin enhanced the immunosuppressive properties of the stem cells by reducing Th17 cell differentiation and NF-κB activation, while upregulating IL-6, NO, and indoleamine-2,3-dioxygenase—an enzyme involved in tryptophan catabolism and T-cell suppression—thereby attenuating pro-inflammatory responses [26].

In human hepatocytes, 5-caffeoylquinic acid, a major chlorogenic acid also present in yerba mate, activated the Nrf2/ARE pathway, upregulated antioxidant enzymes, and protected cells from oxidative stress–induced ROS accumulation and glutathione depletion [29]. Across the included clinical studies, the overall methodological quality ranged from low to high risk of bias, with common issues related to randomization, attrition, and reporting. Detailed domain-level assessments are presented in Supplementary Table S1. Similarly, the in vitro evidence was characterized by predominantly moderate-to-high risk of bias, with recurrent issues related to absence of randomization, lack of blinding, and incomplete reporting of culture conditions and outcome assessment (Supplementary Table S2).

### 3.3. Animal Studies

A total of five animal studies were identified, all employing *Ilex paraguariensis* infusions or non-fractionated extracts in models of adjuvant-induced arthritis or systemic inflammation (Table 2). Doses ranged from 10 to 800 mg/kg, administered orally for periods between a single dose and four weeks. In the adjuvant-induced arthritis model [35], yerba mate reduced paw edema and leukocyte infiltration and restored antioxidant enzyme activities. In models of acute or systemic inflammation, including carrageenan-induced pleurisy [33], nociception and paw edema [32], and DSS-induced colitis [36], treatment decreased leukocyte migration, Th1/Th17 polarization, levels of pro-inflammatory cytokines (IL-6, TNF-α, IFN-γ) and increased IL-10. In aged female rats, supplementation improved bone mineral density and decreased bone oxidative stress markers [34]. Across studies, yerba mate administration generally showed anti-inflammatory and antioxidant effects in vivo, although only one experiment used an arthritis-specific model. The animal studies showed a generally high risk of bias, primarily due to limited reporting of randomization procedures, lack of blinding, and potential uncontrolled confounders across the in vivo models. Detailed SYRCLE assessments are presented in Supplementary Table S3.

## 4. Discussion

Although no clinical trials have directly investigated *Ilex paraguariensis* or its main bioactive components in patients with RA, evidence from human studies in other clinical contexts provides preliminary indications of systemic anti-inflammatory and antioxidant effects that could be relevant to RA.

Randomized controlled trials with yerba mate infusions or standardized extracts have reported reductions in circulating inflammatory biomarkers such as CRP and IL-6 in some populations, together with improvements in redox-related parameters, including increased glutathione level. Similar findings have been observed for isolated compounds—particularly quercetin and rutin—which have been associated with reduction in pro-inflammatory cytokines (IL-6, TNF-α) and oxidative stress parameters across different clinical contexts such as cardiovascular risk, diabetes, and postmenopausal states.

While these results suggest a plausible systemic anti-inflammatory and antioxidant activity of yerba mate and its bioactive components in humans, the available evidence remains limited. The number of human studies identified in this review is small, and those that exist are generally short in duration, involve modest sample sizes, and have been conducted in populations without inflammatory joint disease—thus restricting their direct extrapolation to RA. Moreover, considerable heterogeneity in population characteristics, intervention formats, dosages, and exposure durations further limits the interpretability and generalizability of the findings. Furthermore, the outcomes assessed in these studies are limited to general inflammatory or oxidative biomarkers (e.g., CRP, IL-6, TNF-α), which are not validated RA-specific clinical endpoints and cannot be directly extrapolated to disease activity or joint symptoms in RA.

Several mechanistic investigations further support the notion that yerba mate and its major constituents can modulate oxidative stress, cytokine production, and inflammatory signaling cascades, including the NF-κB, JAK/STAT, and NLRP3 inflammasome pathways.

Moreover, evidence from observational and experimental studies indicates that yerba mate may influence bone metabolism through antioxidant and anti-inflammatory mechanisms. Habitual consumption (>1 L/day) has been associated with higher bone mineral density and reduced osteoporosis prevalence among postmenopausal women [24], while animal data suggest increased OPG expression and decreased RANKL and oxidative stress in bone tissue [34]. Given that dysregulated RANKL/OPG signaling and inflammation-driven bone resorption are central features of RA, these findings provide additional mechanistic support for the potential relevance of yerba mate in attenuating bone loss associated with chronic inflammation.

**Figure 1:**
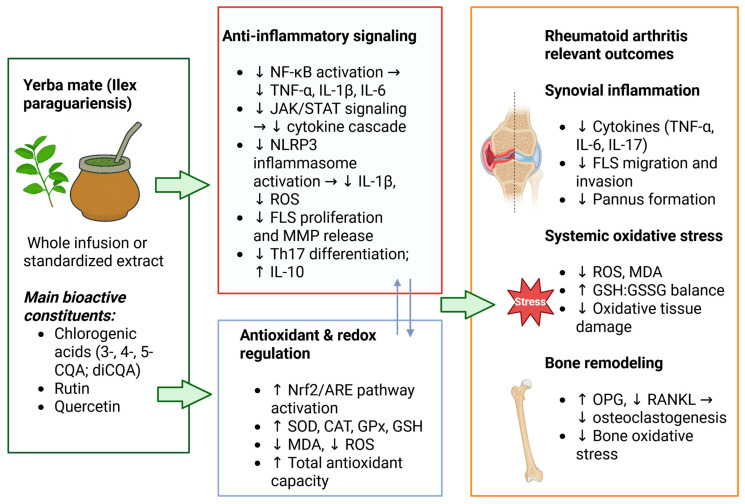
Proposed mechanisms underlying the anti-inflammatory, antioxidant, and bone-protective effects of *Ilex paraguariensis* and its main bioactive constituents in RA. Yerba mate infusions or non-fractioned preparations—rich in chlorogenic acids (3-, 4-, 5-CQA; diCQA), rutin, and quercetin—modulate inflammatory and oxidative pathways relevant to RA. Anti-inflammatory effects include inhibition of NF-κB, JAK/STAT, and NLRP3 signaling, leading to reduced cytokine release (TNF-α, IL-1β, IL-6), reduced FLS proliferation, and decreased Th17 differentiation, together with increased IL-10. Activation of the Nrf2/ARE pathway enhances antioxidant defenses (↑ SOD, CAT, GPx, GSH) and decreases oxidative stress markers (↓ ROS, ↓ MDA), improving the GSH:GSSG redox balance. At the bone level, yerba mate increases OPG and reduces RANKL expression, limiting osteoclastogenesis and oxidative damage. Collectively, these actions may mitigate synovial inflammation, oxidative stress, and inflammation-driven bone remodeling. Abbreviations: CQA, caffeoylquinic acid; diCQA, dicaffeoylquinic acid; NF-κB, nuclear factor kappa-light-chain-enhancer of activated B cells; JAK/STAT, Janus kinase/signal transducer and activator of transcription; NLRP3, nucleotide-binding domain, leucine-rich repeat-containing family, pyrin domain-containing 3; FLS, fibroblast-like synoviocyte; MMP, matrix metalloproteinase; IL, interleukin; TNF-α, tumor necrosis factor-alpha; ROS, reactive oxygen species; MDA, malondialdehyde; SOD, superoxide dismutase; CAT, catalase; GPx, glutathione peroxidase; GSH, reduced glutathione; GSSG, oxidized glutathione; OPG, osteoprotegerin; RANKL, receptor activator of nuclear factor-κB ligand. Legend: ↓ decrease; ↑ increase; → resulting effect.

Evidence from human-cell and animal models suggests that these actions may underlie anti-inflammatory and antioxidant effects relevant to RA pathophysiology, such as immune activation, oxidative imbalance, tissue damage and bone remodeling. The main mechanistic pathways through which yerba mate and its principal bioactive constituents may influence RA pathophysiology are summarized in Figure 1.

Nonetheless, important methodological limitations—including species-specific differences, the intrinsic reductionism of traditional in vitro systems, and the frequent use of supraphysiological concentrations in mechanistic assays—warrant cautious interpretation of these preclinical findings.

Prospectively designed randomized, placebo-controlled trials in patients with RA are warranted to determine clinical efficacy and safety, using RA-specific endpoints (e.g., DAS28, ACR response, RA-specific imaging outputs), adequate treatment duration, and predefined biomarker panels to capture target engagement.

### 4.1. Critical Appraisal of Preclinical Evidence: Species Differences, Limitations of Traditional In Vitro Models, and Supraphysiological Concentrations

Despite offering mechanistic insight, current preclinical models present several limitations that restrict their translational value.

These include interspecies differences, the inherent simplification of traditional in vitro systems, and the reliance on concentrations that may not reflect realistic human systemic exposure.

It is well established that major interspecies differences exist in pharmacokinetics, including absorption, distribution, metabolism, and excretion of xenobiotics, which can markedly alter systemic exposure and biological effects across species [37,38]. In the specific case of yerba mate, several of its major bioactive compounds, such as methylxanthines, quercetin derivatives, and rutin, exhibit inter-species variability in pharmacokinetics and metabolism [39,40,41,42]. Such interspecific differences highlight the limitations of directly extrapolating anti-inflammatory or antioxidant effects observed in rodents or other animal models to humans.

Moreover, major discrepancies exist between human RA and experimental arthritis models in rodents. While adjuvant- or collagen-induced arthritis partially reproduce synovial inflammation and joint destruction, they do not fully capture the chronicity, autoimmune features, and heterogeneity of human RA. Critical aspects such as autoantibody production, FLS behaviour, and systemic comorbidities are often absent or oversimplified, limiting the external validity of findings from animal studies [43].

Given these limitations, it is also noteworthy that the animal studies included in this review—consistent with a well-recognized issue in preclinical research—employed heterogeneous dosing regimens, based in some cases on approximations from traditional human consumption and in others on arbitrarily selected amounts. None provided formal human-equivalent dose calculations or pharmacokinetic justification. Moreover, classical allometric scaling has intrinsic constraints [44,45,46], particularly for phytochemicals characterized by low oral bioavailability, extensive first-pass metabolism, and microbiota-dependent biotransformation, for which allometric extrapolations often fail to predict human systemic exposure. This variability further constrains the translational value of the preclinical evidence, as the systemic exposures achieved in animals may not reflect physiologically attainable levels in humans.

Traditional in vitro systems, typically based on immortalized cell lines or static monocultures, provide only a reductionist view of the RA microenvironment. They fail to capture the dynamic interactions between immune cells, synoviocytes, cartilage, and bone that drive disease progression, and they also lack cross-talk with other organs such as the liver and gut, which play a central role in the metabolism and transformation of bioactive compounds. This is particularly relevant for key yerba mate phytochemicals—such as caffeoylquinic and dicaffeoylquinic acids—which undergo extensive first-pass metabolism, phase II conjugation, and colonic microbial biotransformation in humans in vivo [42,47], resulting in low circulating levels of the parent compounds and the predominance of bioactive metabolites rather than the intact molecules tested in vitro.

In this context, the widespread use of in vitro concentrations that exceed physiologically achievable human exposure further limits the translational value of mechanistic findings, as many experimental doses are substantially higher than the plasma ranges documented in human pharmacokinetic studies. Taken together, these factors indicate that conventional in vitro systems may not accurately reflect the biochemical milieu in which *Ilex paraguariensis* constituents act in vivo.

### 4.2. Future Perspectives and Research Directions

Future research should move beyond classical animal models and reductionist in vitro systems toward human-relevant, mechanistically informative approaches. Advanced experimental platforms such as multicellular organoids and microfluidic organ-on-chip systems can more accurately reproduce the complex architecture and cellular interactions characteristic of the rheumatoid joint, including synovial fibroblasts, immune cells, endothelial components, and bone-resorbing cells [48,49,50]. When coupled with gut- and liver-on-chip models, these technologies also allow investigation of the absorption, first-pass metabolism, and microbiota-mediated biotransformation of orally administered phytochemicals such as those found in *Ilex paraguariensis* [51,52].

Integrating these physiologically based models with multi-omics profiling and in silico simulations may provide deeper insight into the molecular pathways and network-level interactions modulated by complex phytochemical mixtures under conditions that closely mimic human physiology [53].

Importantly, future mechanistic studies should incorporate human pharmacokinetic data to guide dose selection and ensure the use of exposure ranges that are physiologically achievable. In humans, chlorogenic acids undergo limited absorption in their native form and circulate mainly as microbial and phase-II metabolites [47,54,55]; rutin is poorly absorbed as an intact glycoside and contributes chiefly to circulating quercetin conjugates [56]; quercetin itself is extensively metabolized during first-pass processing and appears in plasma predominantly as glucuronidated, sulfated, or methylated derivatives [57,58,59]; in contrast, methylxanthines such as caffeine are efficiently absorbed and reach measurable systemic levels [60]. These well-established pharmacokinetic features indicate that most native phenolic constituents of *Ilex paraguariensis* may exhibit low systemic availability, underscoring the need to test concentrations aligned with documented human exposure rather than extremely high experimental doses. Furthermore, human pharmacokinetic studies using yerba mate infusions or extracts are still lacking, and the systemic exposure achieved after consumption of the complex phytochemical matrix remains insufficiently characterized. Such data are needed to define realistic human exposure ranges and to guide mechanistic experiments toward physiologically relevant concentration windows. Early-phase dose-finding and pharmacokinetic/pharmacometabolomic studies in RA populations will also be essential to quantify systemic exposure to native compounds and derived metabolites, evaluate inter-individual variability (including microbiota-related influences), and link exposure profiles to pharmacodynamic responses.

Equally important is the need to investigate the biological activity of the yerba mate as an intact preparation, rather than focusing exclusively on isolated constituents. Its bioactive potential reflects interactions among multiple co-occurring components, which may modulate each other’s effects. For example, in vitro data indicate that saponins and quercetin can jointly inhibit iNOS and COX-2 expression through NF-κB modulation, whereas the corresponding unfractionated extract shows lower potency, potentially due to competing interactions within the mixture [61]. Therefore, future studies should aim to characterize the integrated biological effects of complete yerba mate preparations—particularly in human-relevant models—so as to capture the full complexity of their mechanistic actions.

**Figure 2:**
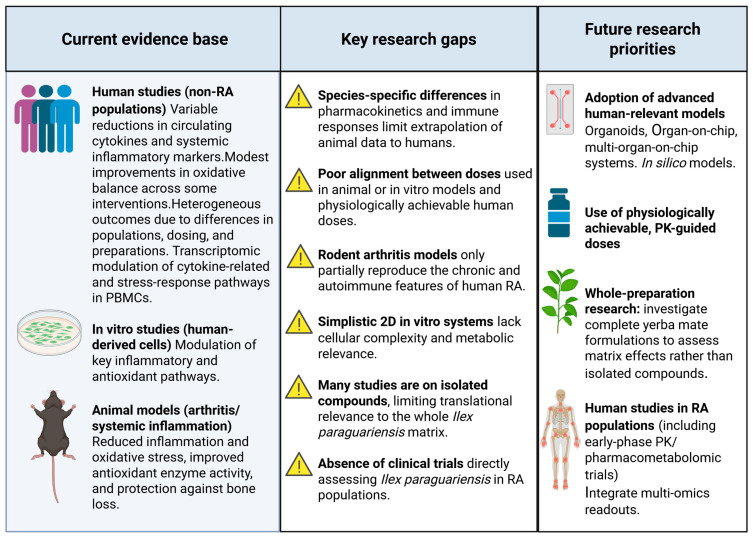
Current evidence and future research priorities for *Ilex paraguariensis* in RA. Evidence of anti-inflammatory and antioxidant activity derives from non-RA human studies and preclinical in vitro and animal models, which offer mechanistic insights but have limited translational relevance due to species differences, non–RA-specific endpoints, and supraphysiological exposures. Future research should prioritize advanced human-relevant models (organoids, organ-on-chip), multi-omics integration, and well-conducted clinical trials in RA populations using yerba mate as a complete, non-fractionated preparation (“whole”) to strengthen clinical translation. Created in BioRender (https://BioRender.com/).

The conceptual framework summarizing current evidence, key research gaps, and future research priorities for *Ilex paraguariensis* in RA is illustrated in Figure 2.

### 4.3. Limitations of the Present Review

This review has several limitations that should be acknowledged.

First, no clinical studies have been conducted specifically in individuals with RA, and all available evidence derives from non-RA human populations, animal models, or in vitro systems. Consequently, the endpoints assessed in human studies are largely non–RA-specific, relying on general inflammatory or oxidative markers rather than disease-relevant outcomes such as joint inflammation, autoantibodies, or validated composite activity scores. This substantially limits the extent to which current mechanistic findings can be directly extrapolated to RA. Second, substantial heterogeneity across study designs, populations, intervention forms, and outcome measures precluded quantitative synthesis and limits comparability among studies. Third, the overall body of evidence is relatively small, spanning a limited number of clinical, animal, and in vitro investigations, many of which involve modest sample sizes or restricted experimental conditions. Fourth, most preclinical data rely on animal models or simplified in vitro systems that may not accurately reproduce human pharmacokinetics, immune mechanisms, or disease complexity, thereby reducing translational applicability. In addition, dosing regimens show substantial variability. Many in vitro studies use supraphysiological concentrations, and doses employed in animal models are often empirical and not informed by human pharmacokinetic data. These factors further complicate the interpretation and translational relevance of mechanistic findings. Fifth, most human trials were characterized by concerns in randomization, blinding, or outcome reporting, as reflected by predominantly moderate-to-high risk-of-bias assessments. Preclinical in vitro studies frequently lacked key methodological safeguards such as randomization, blinding, and standardized outcome assessment, while animal experiments often had unclear or high risk of bias due to insufficient reporting of allocation procedures, randomization, blinding, and housing or experimental conditions. These limitations substantially reduce the internal validity and generalizability of the available findings and further underscore that current evidence should be interpreted as preliminary.

Finally, publication bias cannot be excluded, as studies reporting null or negative results are less likely to be published. Despite these limitations, the present review integrates the best up-to-date available human, in vitro, and animal evidence to inform mechanistic processes potentially relevant to RA, thereby providing an improved foundation for future disease-specific research.

## 5. Conclusions

The available evidence suggests that yerba mate may exert anti-inflammatory and antioxidant effects that could be mechanistically relevant to RA. Several human studies conducted in metabolic, cardiovascular, or general inflammatory settings have shown improvements in oxidative balance and modulation of inflammatory pathways. However, no clinical trials to date have evaluated yerba mate in patients with RA, and the current evidence is insufficient to support its use for disease management. To address these gaps, well-designed human studies specifically targeting RA are warranted. Future research should also prioritize advanced, human-relevant platforms—including organoids, organ-on-chip systems, and in silico models—capable of capturing complex immune and joint-specific processes more accurately than animal or simplistic in vitro systems. Such rigorously conducted, human-centered studies will be essential to determine the true translational potential of yerba mate and its bioactive constituents in RA.

## Figures and Tables

**Table 1:** Human clinical and in vitro studies on yerba mate and isolated constituents with potential RA-relevant outcomes.

Study Design	Population/Cells	Intervention (Mate or Compound)	Outcomes Assessed	Main Findings	Risk of Bias	References
Randomized, double-blind, placebo-controlled crossover trial	Adults at cardiovascular risk, 45–65 y (*n* = 34 men)	Encapsulated dry mate extract (580 mg caffeoylquinic acids/day, 4 weeks)	CRP, IL-6	↓ CRP (−50%) 0.50 ± 0.18 vs. 0.60 ± 0.25 mg/dL; *p* < 0.05 and ↓ IL-6 (−19%) 1.71 ± 0.26 vs. 1.39 ± 0.17 pg/mL only in higher-risk group (*p* < 0.05)	Some concerns (RoB 2)	[14]
Randomized, double-blind, placebo-controlled, crossover clinical trial	Adults with HIV/AIDS on ART ≥6 months, virally suppressed (*n* = 92)	Yerba mate 3 g/day soluble preparation vs. mate-placebo for 15 days	hs-CRP; fibrinogen; lipid profile (including HDL-c); white blood cell indices; oxidative stress (TBARS)	No significant changes in hs-CRP, fibrinogen, or TBARS vs. baseline/placebo (all *p* > 0.05)	High (RoB 2)	[15]
Randomized, controlled, single-blind, crossover trial	Adults, non-habitual yerba mate consumers, 18–55 y (*n* = 52 completers: 25 normocholesterolemic; 27 hypercholesterolemic)	Yerba mate tea (roasted), 3 servings/day (each 3 g sachet infused 5 min in 150 mL; ~9 g leaves/day). Estimated intake: ~666 mg/day (poly)phenols; ~66 mg/day caffeine. Control: decaffeinated, polyphenol-free isotonic drink, 3×/day. Diet: restriction of other polyphenol/methylxanthine sources.	Inflammatory cytokines (IL-1β, -2, -4, -5, -6, -7, -8, -10, -12, -13), TNF-α, IFN-γ; hsCRP; lipid peroxidation (MDA)	Broad ↓ of ILs (all *p* < 0.001–0.003), TNF-α ~40–50% reduction (*p* < 0.001), IFN-γ ~40–50% reduction (*p* < 0.001); hsCRP ↓ in both groups −55%; *p* = 0.031; MDA ↓	High (RoB 2)	[16]
Pilot, two-phase crossover study (self-controlled, no placebo)	Healthy adults, men, 25 ± 3 y (*n* = 9)	Soluble mate tea (1 g/200 mL, 3×/day, 8 days)	Leukocyte p47phox; serum TNF-α, IL-6, IL-1β; plasma phenols; GSH, GSSG, GSH:GSSG ratio	↓ p47phox (−22%; *p* = 0.030); ↓ TNF-α (−56%; *p* = 0.010); ↓ IL-6 (−52%; *p* = 0.012); ↑ plasma phenols (+30%; *p* = 0.004); ↑ GSH:GSSG ratio (+98%; *p* = 0.015); improved redox balance (↑GSH + 16.5%, *p* = 0.049; ↓GSSG −34%, ns).	Some concerns (RoB2)	[17]
Randomized crossover trial	Healthy adults, men (*n* = 12)	Mate tea (3 × 200 mL/day, 11 days; ~890 mg polyphenols) vs. water	Eccentric exercise; isometric strength; plasma phenolics; GSH, GSSG, GSH:GSSG ratio; LOOH	↑ plasma phenolics (*p* = 0.008); Preserved GSH (mate prevented the 48–72 h decline seen in control; *p* = 0.002); faster strength recovery at 24 h (+8.6%; *p* = 0.009); no effects on GSSG, GSH:GSSG ratio, or LOOH.	High (RoB2)	[18]
Randomized, double-blind, crossover trial	Adults, men, 45–65 y, ≤1 metabolic syndrome criterion (*n* = 34)	Standardized yerba mate extract (2250 mg/day, ~581 mg CQA), 4 weeks vs. placebo	PBMC transcriptomics; inflammatory biomarkers (CRP, IL-6); NF-κB/MAPK/PI3K-Akt pathways	Modulated PBMC gene expression: 2635 DEGs (↑ 2385 protein-coding; ↓ 244 lncRNA; ↓ 6 miRNA). Subgroup: ↓ CRP (*p* = 0.031) and ↓ IL-6 (*p* < 0.001). Pathways: modulation of cytokine–cytokine receptor interaction, chemokine, MAPK, and PI3K-Akt signalling pathways.	High (RoB 2)	[19]
Randomized, double-blind, placebo-controlled trial	Adults with type 2 diabetes mellitus (*n* = 50)	Rutin supplement, 500 mg/day, 3 months	IL-6, MDA, TAC	↓ IL-6 (−7.1 pg/mL; *p* = 0.002); ↓ MDA (−3.6 µM; *p* < 0.001); ↑ TAC (+0.16 mM; *p* < 0.001).	Low (RoB 2)	[20]
Randomized, double-blind, placebo-controlled parallel-group trial	Post–myocardial infarction adults, 35–65 y (*n* = 88)	Quercetin 500 mg/day (oral tablets, 8 weeks)	hs-CRP, IL-6, TNF-α, TAC	↑ TAC (*p* < 0.001); ↓ TNF-α (within-group *p* = 0.009, not significant vs. placebo); no effect on hs-CRP or IL-6.	Some concerns (RoB 2)	[21]
Randomized, double-blind, placebo-controlled trial	Healthy post-menopausal women (*n* = 33)	Quercetin 500 mg/day (oral tablets, 90 days)	IL-6, TNF-α, CRP	↓ IL-6 (*p* = 0.045) and ↓ TNF-α (*p* = 0.021) vs. placebo; no effect on CRP	Some concerns (RoB 2)	[22]
Randomized, double-blind, placebo-controlled trial	Adults undergoing coronary artery bypass surgery (*n* = 97)	Quercetin, 500 mg twice daily, 2 days pre-surgery → hospital discharge (max 7 days)	hsCRP; NO-dependent endothelial functions; SnRNA-seq; Olink 384-protein inflammation panel	↓ hs-CRP in men (*p* < 0.05; group × time *p* = 0.025); ↓ IL-6/JAK-STAT3 and ↓ TNF-α/NF-κB pathways; ↑ NO-dependent endothelial relaxation in men (*p* < 0.05); DEPs in men defined by FDR < 0.05	Some concerns (RoB 2)	[23]
Observational case–control study	Postmenopausal women consuming ≥1 L/day YM (*n* = 153) vs. non-consumers (*n* = 147)	Habitual yerba mate consumption	BMD (DXA), cortical/trabecular vBMD, osteoporosis diagnosis, fragility fractures	↑ Total hip BMD (+8%; *p* < 0.0001); ↑ cortical & trabecular vBMD (all *p* < 0.0001); ↓ osteoporosis prevalence (3.3% vs. 10.9%; OR 0.276, *p* = 0.012); ↓ low-impact fractures (5.9% vs. 12.9%; OR 2.197, *p* = 0.046).	Serious (ROBINS-I)	[24]
In vitro mechanistic study	Human RA-FLSs	Quercetin (0, 10, 20 or 30 μM)	Cell migration and invasion, F-actin expression, miR-146a and GATA6 levels	↑miR-146a and ↓ GATA6, leading to ↓ F-actin expression and suppression of RA-FLS migration and invasion	High (OHAT)	[25]
In vitro	Human PBMCs from healthy donors (TNF-α/IFN-γ–stimulated)	Quercetin (10 μM) pretreatment of hUCMSCs before coculture with activated PBMCs	PBMC proliferation; Th17 cell proportion; expression of TLR-3, *p*-AKT, *p*-IκB in hUCMSCs; secretion of IL-6, NO, and IDO	Quercetin enhanced the immunosuppressive effect of hUCMSCs → ↓PBMC proliferation; ↓ Th17 cells; ↑ TLR-3; ↓ *p*-AKT and ↓ *p*-IκB; ↑IL-6, ↑ NO, ↑ IDO	Some Concerns (OHAT)	[26]
In vitro	Human hepatoma HepG2 cells	YMPE, metabolites (DHCA, DHFA)	CV, LDH leakage, ROS, GSH, GPx, GR, MDA, protein carbonyls	YMPE and DHCA ↓ROS, ↓ LDH, ↓ MDA, ↓ carbonyls, ↑ GSH, normalized GPx/GR; DHFA partially effective;	Low (OHAT)	[27]
In vitro mechanistic study	Human RA-FLSs	Quercetin (50 nmol/L, pretreatment 2 h)	IL-1β, IL-6, IL-8; XIST, miR-485, PSMB8 expression	↓ Inflammatory cytokines (IL-1β, IL-6, IL-8) and ↓ XIST expression in TNF-α–stimulated RA-FLSs; restored miR-485; suppressed PSMB8 upregulation; anti-inflammatory effect lost when PSMB8 silenced	High (OHAT)	[28]
In vitro	Human hepatocyte cell line (HepG2)	5-Caffeoylquinic acid (5-CQA), 10–100 μM	ROS, GSH, Nrf2 nuclear translocation, ARE activity, HO-1, GCL, NQO1, Sestrin2 expression ↑ Nrf2 activation and downstream antioxidant enzymes (HO-1, GCL, NQO1, Sestrin2)	↓ ROS production and prevention of GSH depletion under oxidative stress; protective effect abolished by Nrf2 knockout or inhibitor	High (OHAT)	[29]
In vitro	Human endothelial EA.hy926 cells	YMPE prepared from commercial yerba mate leaves and stems (1–50 µg/mL).	ROS, GSH, GPx, GR, protein carbonyls, eNOS levels	↓ ROS; ↑ GSH; ↓ GPx, ↓ GR overactivation; ↓ protein carbonyls; ↑ eNOS → prevention of TNF-α–induced oxidative stress and endothelial dysfunction	High (OHAT)	[30]
In vitro + in silico docking	Human THP-1 macrophages	YME prepared from commercial pure leaf yerba mate (1–500 μg/mL (range), with 15 μg/mL used in efficacy experiments).	Cell viability; NO, ROS; NLRP3 inflammasome activation; gene expression; docking of chlorogenic acid and rutin to NLRP3 (MCC950 binding site)	Suppression of NLRP3 inflammasome activation in macrophages, ↓ NO and ROS, and attenuated pro-inflammatory responses; chlorogenic acid and rutin showed high predicted affinity for NLRP3 inhibition	High (OHAT)	[31]

Abbreviations: BMD, Bone Mineral Density; DXA, Dual-energy X-ray Absorptiometry; sBMD, Surface Bone Mineral Density (cortical); vBMD, Volumetric Bone Mineral Density (trabecular); NPSHs, Non-Protein Thiols; TBARSs, Thiobarbituric Acid Reactive Substances; t-BHP, tert-Butyl Hydroperoxide; eNOS, Endothelial Nitric Oxide Synthase; GPx, Glutathione Peroxidase; GR, Glutathione Reductase; GSH, Reduced Glutathione; ROS, Reactive Oxygen Species; CV, Cell Viability; DHCA, Dihydrocaffeic Acid; DHFA, Dihydroferulic Acid; LDH, Lactate Dehydrogenase; MDA, Malondialdehyde; YME, Yerba Mate Aqueous Extract; YMPE, Yerba Mate Phenolic Extract; RA-FLSs, Rheumatoid Arthritis Fibroblast-like Synoviocytes; XIST, X-inactive Specific Transcript (long non-coding RNA); PSMB8, Proteasome Subunit Beta 8; GATA6, GATA-binding Protein 6; PBMCs, Peripheral Blood Mononuclear Cells; hUCMSCs, Human Umbilical Cord Mesenchymal Stem Cells; TNF-α, Tumor Necrosis Factor-alpha; IFN-γ, Interferon-gamma; TLR-3, Toll-Like Receptor 3; *p*-AKT, Phosphorylated Protein Kinase B; *p*-IκB, Phosphorylated Inhibitor of kappa B; IL-6, Interleukin-6; NO, Nitric Oxide; IDO, Indoleamine 2,3-dioxygenase; Th17, T helper 17 cells; MDA, Malondialdehyde; TAC, Total Antioxidant Capacity. Legend: ↓ decrease; ↑ increase; → resulting effect.

**Table 2:** Animal Studies Evaluating Yerba Mate in Models Potentially Relevant to RA.

Species/Model	Intervention	Dose/Duration	Outcomes Assessed	Main Findings	Risk of bias	References
Mice; formalin-induced orofacial nociception, writhing test, paw formalin test, carrageenan-induced paw edema	*Ilex paraguariensis* aqueous leaf infusion (prepared from 100 g dried leaves infused in 1 L water at 85 °C for 10 min, filtered, dried at 40 °C, and reconstituted before administration).	Oral, 1–3 g/kg, acute administration. Dose derived from habitual human mate consumption (light/moderate/heavy drinkers); no PK or formal human-equivalent dose calculation reported.	Nociception, paw edema, mechanistic pathways	↓ Writhing; ↓ Orofacial pain (formalin test, both phases); ↔ Paw edema; ↔ Paw formalin test; effect blocked by α1-adrenoceptor antagonist → noradrenergic pathway involvement	High (SYRCLE)	[32]
Female Swiss mice, carrageenan-induced pleurisy	Hydroethanolic leaf extract of *Ilex paraguariensis* (CE) and fractions (BF, ARF), prepared by turboextraction of lyophilized leaves in 20% ethanol (1:5 m/v, 5 min); isolated compounds: Caf, Rut, CGA.	CE 10–50 mg/kg; BF and ARF 0.1–10 mg/kg; Caf 0.1–5 mg/kg; Rut 0.01–1 mg/kg; CGA 0.01–1 mg/kg; all given orally 0.5 h before pleurisy induction	Leukocyte and neutrophil migration, exudate concentration, MPO and ADA activity, NOx levels, cytokine levels (IL-6, IL-17A, IFN-γ, TNF-α, IL-10), lung histology, NF-κB p65 phosphorylation	↓ Leukocyte and neutrophil influx; ↓ exudation, MPO, ADA, NOx; ↓ IL-6, IL-17A, IFN-γ, TNF-α; ↑ IL-10; improved lung histology; ↓ NF-κB p65 phosphorylation → overall attenuation of Th1/Th17 polarization	High (SYRCLE)	[33]
Female Wistar rats, “perimenopausal” (16 months);	Mate tea instant powder (commercial preparation; reconstituted in water, 0.05 g/mL; plant part not specified).	20 mg/kg/day; Dose stated by authors as equivalent to human consumption of ~300 mL/day of mate tea and previously used in their earlier work; 4 weeks.	Areal bone mineral density (aBMD), trabecular area, osteocyte number, plasma TRAP (osteoclast activity) and ALP (osteoblast activity), bone MDA (oxidative stress marker), immunohistochemistry for RANKL, OPG, SOD2;	Increased aBMD, trabecular area, and osteocyte number; ↓ TRAP, RANKL, SOD2; ↑ OPG; ↓ bone MDA; suggesting reduced oxidative stress and inhibition of osteoclastogenesis via RANKL-dependent pathway;	High (SYRCLE)	[34]
Rat, adjuvant-induced arthritis	Hot-water aqueous extract of *Ilex paraguariensis* leaves (traditional chimarrão; 85 g leaves/1.5 L water, 5 min; lyophilized)	Oral, 400 or 800 mg/kg/day, 23 days	ROS, oxidative damage, antioxidant enzymes (SOD, CAT, GPx, GR, XO), GSH/GSSG, paw edema, leukocyte infiltration	Improved antioxidant status (↑ GSH, restored enzyme activity, ↓ ROS, ↓ damage); reduced paw swelling and inflammatory infiltration	High (SYRCLE)	[35]
Mouse; DSS-induced colitis model	Commercial leaf dry extract of *Ilex paraguariensis*, dissolved in hot water (60 °C; 1 g in 8 mL), filtered (0.2 µm)	Oral gavage, 0.025 g/mouse; 7-day pretreatment + DSS (3%) for 7 days	Inflammatory macrophage infiltration and polarization (F4/80^+^, CD206^+^, CD301^+^); modulation of gut inflammatory milieu	↓ Pro-inflammatory macrophage infiltration; ↑ M2 (anti-inflammatory) macrophage polarization in colon;	High (SYRCLE)	[36]

Abbreviations: ADA, Adenosine Deaminase; ARF, Aqueous Residual Fraction; BF, Butanolic Fraction; Caf, Caffeine; CE, Crude Extract; CGA, Chlorogenic Acid; DEGs, Differentially Expressed Genes; DEPs, Differentially Expressed Proteins; DSS, dextran sodium sulfate; MPO, Myeloperoxidase; NOx, Nitric Oxide species; Rut, Rutin; Th, T helper; aBMD, areal Bone Mineral Density; TRAP, Tartrate-Resistant Acid Phosphatase; ALP, Alkaline Phosphatase; MDA, Malondialdehyde; RANKL, Receptor Activator of Nuclear Factor κB Ligand; OPG, Osteoprotegerin; SOD2, Superoxide Dismutase 2. Legend: **↓** decrease; ↑ increase; ↔ no change; → resulting effect.

## Data Availability

All data analyzed in this study are derived from previously published articles cited throughout this manuscript. The full list of references is provided in the References section.

## Supplementary Materials

The following supporting information can be downloaded at: https://www.mdpi.com/article/10.3390/nu17243853/s1, Supplementary File S1: Prisma_FlowDiagram_Cassotta_et_al; Supplementary Table S1: Risk of Bias Assessment for Human Studies; Supplementary Table S2: Risk of bias for in vitro studies. Risk of Bias assessment: OHAT tool for in vitro studies.; Supplementary Table S3: Risk of bias for animal studies. Risk of Bias assessment: SYRCLE tool. Ref. [62] is cited in Supplementary Materials file.

## Abbreviations

The following abbreviations are used in this manuscript:

ADA	Adenosine Deaminase
ACR	American College of Rheumatology
ALP	Alkaline Phosphatase
aBMD	areal Bone Mineral Density
ARF	Aqueous Residual Fraction
BF	Butanolic Fraction
BMD	Bone Mineral Density
Caf	Caffeine
CAT	Catalase
CE	Crude Extract
CGA	Chlorogenic Acid
CRP	C-Reactive Protein
CQA	Caffeoylquinic Acid
CV	Cell Viability
DAS28	Disease Activity Score in 28 joints
DEGs	Differentially Expressed Genes
DEPs	Differentially Expressed Proteins
DHCA	Dihydrocaffeic Acid
DHFA	Dihydroferulic Acid
diCQA	Dicaffeoylquinic Acid
DSS	Dextran Sodium Sulfate
DXA	Dual-energy X-ray Absorptiometry
eNOS	Endothelial Nitric Oxide Synthase
ESR	Erythrocyte Sedimentation Rate
FLS	Fibroblast-like Synoviocyte
GATA6	GATA-binding Protein 6
GPx	Glutathione Peroxidase
GR	Glutathione Reductase
GSH	Reduced Glutathione
GSSG	Oxidized Glutathione
hUCMSCs	Human Umbilical Cord Mesenchymal Stem Cells
IDO	Indoleamine 2,3-Dioxygenase
IFN-γ	Interferon-gamma
IL	Interleukin
IL-6	Interleukin-6
JAK/STAT	Janus Kinase/Signal Transducer and Activator of Transcription
LDH	Lactate Dehydrogenase
MDA	Malondialdehyde
MMP	Matrix Metalloproteinase
MPO	Myeloperoxidase
NF-κB	Nuclear Factor Kappa-light-chain-enhancer of Activated B Cells
NLRP3	Nucleotide-binding Domain, Leucine-rich Repeat-containing Family, Pyrin Domain-containing 3
NO	Nitric Oxide
NOx	Nitric Oxide Species
NPSH	Non-Protein Thiols
OPG	Osteoprotegerin
*p*-AKT	Phosphorylated Protein Kinase B
PBMCs	Peripheral Blood Mononuclear Cells
*p*-IκB	Phosphorylated Inhibitor of Kappa B
PSMB8	Proteasome Subunit Beta 8
RANKL	Receptor Activator of Nuclear Factor κB Ligand
RA-FLSs	Rheumatoid Arthritis Fibroblast-like Synoviocytes
ROS	Reactive Oxygen Species
Rut	Rutin
sBMD	Surface Bone Mineral Density (cortical)
SOD	Superoxide Dismutase
SOD2	Superoxide Dismutase 2
TAC	Total Antioxidant Capacity
TBARS	Thiobarbituric Acid Reactive Substances
Th	T Helper
Th17	T Helper 17 Cells
TLR-3	Toll-Like Receptor 3
TNF-α	Tumor Necrosis Factor-alpha
t-BHP	tert-Butyl Hydroperoxide
TRAP	Tartrate-Resistant Acid Phosphatase
vBMD	Volumetric Bone Mineral Density (trabecular)
XIST	X-inactive Specific Transcript (long non-coding RNA)
YME	Yerba Mate Aqueous Extract
YMPE	Yerba Mate Phenolic Extract

## Appendix A

### Appendix A.1. Search Strings for Pubmed

(“*Ilex paraguariensis*” [MeSH Terms] OR (“ilex” [All Fields] AND “paraguariensis”[All Fields]) OR “*Ilex paraguariensis*” [All Fields] OR (“yerba” [All Fields] AND “mate” [All Fields]) OR “yerba mate” [All Fields] OR (“*Ilex paraguariensis*” [MeSH Terms] OR (“ilex” [All Fields] AND “paraguariensis” [All Fields]) OR “*Ilex paraguariensis*” [All Fields])) AND (2015:2025 [pdat])

(“*Ilex paraguariensis*” [MeSH Terms] OR (“ilex” [All Fields] AND “paraguariensis” [All Fields]) OR “*Ilex paraguariensis*” [All Fields] OR (“yerba” [All Fields] AND “mate” [All Fields]) OR “yerba mate” [All Fields] OR (“*Ilex paraguariensis*” [MeSH Terms] OR (“ilex” [All Fields] AND “paraguariensis” [All Fields]) OR “*Ilex paraguariensis*” [All Fields])) AND (“arthritis” [MeSH Terms] OR “arthritis” [All Fields] OR “arthritides” [All Fields] OR “polyarthritides” [All Fields])

((“chlorogenic acid” [Supplementary Concept] OR “chlorogenic acid” [All Fields] OR “chlorogenic acid” [MeSH Terms] OR (“chlorogenic” [All Fields] AND “acid” [All Fields]) OR (“quercetin” [Supplementary Concept] OR “quercetin” [All Fields] OR “quercetin” [MeSH Terms] OR “quercetin s” [All Fields] OR “quercetine” [All Fields] OR “quercetins” [All Fields]) OR (“ursolic acid” [Supplementary Concept] OR “ursolic acid” [All Fields] OR “ursolic acid” [MeSH Terms] OR (“ursolic” [All Fields] AND “acid” [All Fields])) OR (“rutin” [Supplementary Concept] OR “rutin” [All Fields] OR “rutin” [MeSH Terms] OR “rutins” [All Fields])) AND (“arthritis, rheumatoid” [MeSH Terms] OR (“arthritis” [All Fields] AND “rheumatoid” [All Fields]) OR “rheumatoid arthritis” [All Fields] OR (“rheumatoid” [All Fields] AND “arthritis” [All Fields]))) AND (2020:2025 [pdat])

((“caffeoylquinic acid” [All Fields] OR “3-caffeoylquinic acid” [All Fields] OR “4-caffeoylquinic acid” [All Fields] OR “5-caffeoylquinic acid” [All Fields] OR “3,4-dicaffeoylquinic acid” [All Fields] OR “3,5-dicaffeoylquinic acid” [All Fields] OR “4,5-dicaffeoylquinic acid” [All Fields]) AND (“inflammation” [MeSH Terms] OR (“inflammatories” [All Fields] OR “inflammatory” [All Fields]) OR “oxidative stress” [All Fields] OR (“cytokin” [All Fields] OR “cytokine s” [All Fields] OR “cytokines” [Supplementary Concept] OR “cytokines” [All Fields] OR “cytokine” [All Fields] OR “cytokines” [MeSH Terms] OR “cytokinic” [All Fields] OR “cytokins” [All Fields]) OR “arthritis” [All Fields] OR “rheumatoid arthritis” [All Fields])) AND (2020:2025 [pdat])

### Appendix A.2. Search Strings for SciELO

“*Ilex paraguariensis*” OR “yerba mate”

“Chlorogenic acid” AND “rheumatoid arthritis”

“Rutin” AND “rheumatoid arthritis”

“Quercetin” AND “rheumatoid arthritis”

“Ursolic acid” AND “rheumatoid arthritis”

### Appendix A.3. Search Strings for LILACS

“*Ilex paraguariensis*” OR “yerba mate” AND db:(“LILACS”) AND (year_cluster:[2015 TO 2025]) AND instance:”lilacsplus”

(“*Ilex paraguariensis*” OR “yerba mate”) AND (“rheumatoid arthritis” OR “inflammation” OR “oxidative stress”) AND db:(“LILACS”) AND (year_cluster:[2015 TO 2025]) AND instance: ”lilacsplus”
