# Supplementary material for: Yerba Mate (Ilex paraguariensis) and Rheumatoid Arthritis: A Systematic Review of Mechanistic and Clinical Evidence
Source: Nutrients. 2025 Dec 10;17(24):3853. doi: 10.3390/nu17243853 (PMC12735931; doi:10.3390/nu17243853)
Supplement: Supplementary file 1 [file nutrients-17-03853-s001.zip › nutrients-4012191-supplementary.pdf]

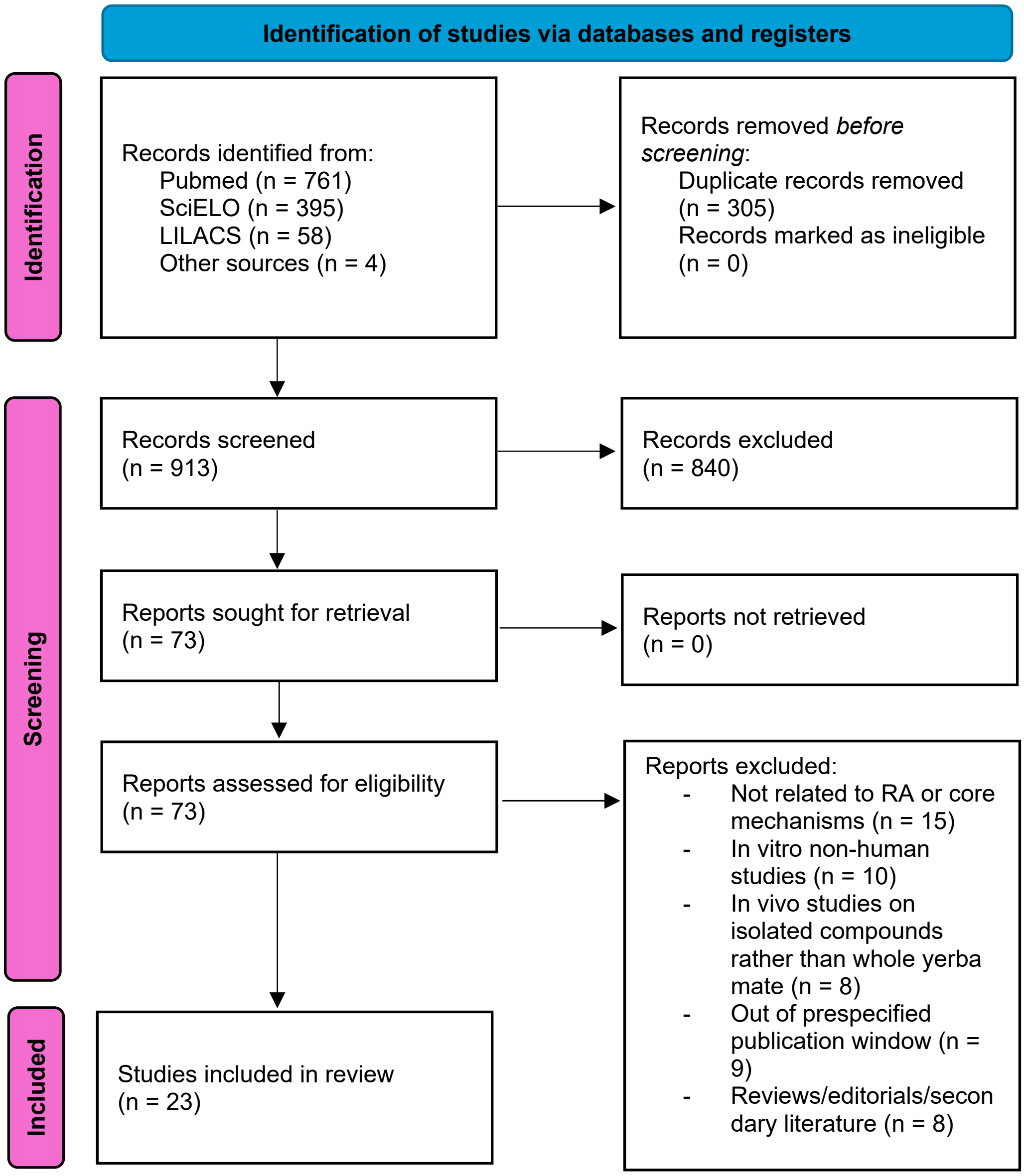

PRISMA 2020 flow diagram illustrating the identification, screening, eligibility assessment, and inclusion process of studies investigating Ilex paraguariensis and its bioactive constituents in relation to RA–relevant mechanisms.

Source: Page MJ, et al. BMJ 2021;372:n71. doi: 10.1136/bmj.n71.

This work is licensed under CC BY 4.0. To view a copy of this license, visit https://creativecommons.org/licenses/by/4.0/

Supplementary Table 1. Risk of Bias Assessment for Human Studies

Study Reference	Design	Bias Tool	D1	D2	D3	D4	D5	D6	D7	Overall RoB	Support for Judgement (Key Points)
Gebara et al., 2021 [1]	RCT, Crossover	RoB 2	Some Concerns	Low	Low	Low	Some Concerns			Some Concerns	D1: Randomization mentioned but method and allocation concealment not described. D5: Protocol registered, but key inflammatory results (CRP, IL-6) come from a post-hoc subgroup analysis.
Petrilli et al., 2016 [2]	RCT, Crossover	RoB 2	Some Concerns	Low	High	Low	Low			High	D1: Randomization mentioned but method and allocation concealment not described. D3: High dropout rate (40.3%). Analysis likely per-protocol, not ITT.
Bravo et al., 2025 [3]	RCT, Crossover, Single-Blind	RoB 2	Some Concerns	High	Low	Low	Some Concerns			High	D1: Participants randomly assigned 1:1, but the method for sequence generation and allocation concealment is not described. D2: Study was single-blind (participants only), high risk of performance bias as personnel were not blinded. D5: Trial was retrospectively registered.
Panza et al., 2016 [4]	RCT, Crossover	RoB 2	Some Concerns	Some Concerns	Low	Some Concerns	Some Concerns			Some Concerns	D1: Randomization mentioned but specific method and allocation concealment not clearly described. D2: No placebo control used (water vs. active treatment), participants likely not blinded to intervention. D3: Complete data for all 12 participants, no attrition reported. D4: Objective outcomes (muscle strength, biochemical markers) but potential bias in subjective effort during strength tests. D5: Protocol registered (RBR-5pj5bb) but emphasis on post-hoc analysis of recovery rate rather than primary strength outcomes.
Panza et al., 2019 [5]	Pilot Study	RoB 2	Some Concerns	Some Concerns	High	Low	Some Concerns			High	D1: No randomization; D2: No placebo/blinding; D3: Small sample (n=9); D5: No protocol
Ruskovska et al., 2023 [6]	RCT, Crossover	RoB 2	Some Concerns	Low	High	Low	Some Concerns			**High**	**D1:** Randomization was performed by an "independent person", but the specific method (e.g., computer-generated) and allocation concealment mechanism are not described. **D3:** High rate of missing outcome data in the nutrigenomic analysis. From an initial n=12 selected for this part, 4 samples

Study Reference	Design	Bias Tool	D1	D2	D3	D4	D5	D6	D7	Overall RoB	Support for Judgement (Key Points)
											were excluded (2 for low RNA quality, 2 for poor hybridization), leaving n=8 for the final analysis. The authors do not provide a rationale for why these specific samples failed or an analysis to show that their exclusion did not introduce bias. **D5:** The study protocol was registered. However, the primary outcomes for the main clinical trial (NCT02789722) were clinical biomarkers, while this paper reports a nutrigenomic analysis on a sub-sample. There is a high risk of selective reporting from the vast microarray dataset, where a very large number of genes were tested, and results were focused on significant findings without a pre-specified plan for this omic analysis.
Bazyar et al., 2023 [7]	RCT, Parallel	RoB 2	**Low**	**Low**	**Low**	**Low**	**Low**			**Low**	**D1:** Computer-generated randomization (block size 4) with allocation concealment (sealed codes A/B). **D2:** Double-blind, placebo-controlled. Identical capsules for supplement and placebo. Adherence monitored and >90% consumption required. **D3:** Low attrition (4/50, 8%). Reasons provided (COVID, withdrawal). ITT analysis performed. **D4:** Objective laboratory outcomes (glycemic, lipid, inflammatory markers). Laboratory technician was blinded. **D5:** Trial was registered (IRCT20151128025274N5), and all pre-specified outcomes in the methods appear to have been reported.
Dehghani et al., 2021 [8]	RCT, Parallel-group	RoB 2	Some Concerns	Low	Low	Low	Some Concerns			Some Concerns	D1: Randomization was performed using permuted block randomization via an online system. However, the method of allocation concealment is not explicitly described. D5: The trial was registered. All primary outcomes are reported, but there is selective emphasis on significant findings in secondary outcomes (QOL dimensions) without a pre-specified statistical plan for multiple comparisons.
Bailly et al., 2025 [9]	RCT, Parallel-group	RoB 2	Some Concerns	Low	Low	Low	Some Concerns			Some Concerns	D1: Randomization was performed by a third party using a permuted block method. However, the method of allocation concealment is not explicitly described. D5: The trial was registered. All pre-specified outcomes are reported, but the abstract selectively emphasizes the significant positive

Study Reference	Design	Bias Tool	D1	D2	D3	D4	D5	D6	D7	Overall RoB	Support for Judgement (Key Points)
											findings for bone and inflammatory markers while downplaying the null results for body composition and physical function.
Mury et al., 2025 [10]	RCT, Parallel-group	RoB 2	Low	Low	Low	Low	Some Concerns			Some Concerns	D1: Computer-generated randomization by independent biostatistician with allocation concealment. D2: Double-blind with matched placebo, intention-to-treat analysis. D3: Low dropout rate (7/111), balanced between groups. D4: Objective outcomes (lab measures, snRNA-seq) with blinding. D5: Trial registered (NCT04907253), all pre-specified outcomes reported, but extensive exploratory sex-subgroup analyses and proteomic/transcriptomic findings without pre-specified analysis plan for these exploratory endpoints.
Brun et al., 2025 [11]	Cross-sectional observational	ROBINS-I	Serious	Moderate	Moderate	Low	Low	Low	Low	Serious	D1: Unmeasured confounders (activity, diet). D2: Convenience sample. D3: Self-reported exposure. D4-7: No major concerns.

Legend – RoB 2 Domains (for RCTs):

- **D1:** Bias arising from the randomization process.
- **D2:** Bias due to deviations from intended interventions.
- **D3:** Bias due to missing outcome data.
- **D4:** Bias in measurement of the outcome.
- **D5:** Bias in selection of the reported result.
- **Judgement:** Low / Some Concerns / High

Legend – ROBINS-I Domains (for observational studies):

- **D1:** Bias due to confounding.
- **D2:** Bias in selection of participants.
- **D3:** Bias in classification of interventions.
- **D4:** Bias due to deviations from intended interventions.

- **D5:** Bias due to missing data.
- **D6:** Bias in measurement of outcomes.
- **D7:** Bias in selection of the reported result.
- **Judgement:** Low / Moderate / Serious / Critical

Bibliography

1. Gebara, K.S.; Gasparotto Junior, A.; Palozi, R.A.C.; Morand, C.; Bonetti, C.I.; Gozzi, P.T.; de Mello, M.R.F.; Costa, T.A.; Cardozo Junior, E.L. A Randomized Crossover Intervention Study on the Effect a Standardized Maté Extract (Ilex Paraguariensis A. St.-Hil.) in Men Predisposed to Cardiovascular Risk. *Nutrients* **2020**, *13*, 14, doi:10.3390/nu13010014.

2. Petrilli, A.A.; Souza, S.J.; Teixeira, A.M.; Pontilho, P.M.; Souza, J.M.P.; Luzia, L.A.; Rondó, P.H.C. Effect of Chocolate and Yerba Mate Phenolic Compounds on Inflammatory and Oxidative Biomarkers in HIV/AIDS Individuals. *Nutrients* **2016**, *8*, 132, doi:10.3390/nu8050132.

3. Bravo, L.; Martínez-López, S.; Sierra-Cinos, J.L.; Mateos, R.; Sarriá, B. Yerba Mate (Ilex Paraguariensis St. Hill.) Tea May Have Cardiometabolic Beneficial Effects in Healthy and At-Risk Subjects: A Randomized, Controlled, Blind, Crossover Trial in Nonhabitual Consumers. *Mol Nutr Food Res* **2025**, *69*, e70065, doi:10.1002/mnfr.70065.

4. Panza, V.P.; Diefenthaeler, F.; Tamborindeguy, A.C.; Camargo, C. de Q.; de Moura, B.M.; Brunetta, H.S.; Sakugawa, R.L.; de Oliveira, M.V.; Puel, E. de O.; Nunes, E.A.; et al. Effects of Mate Tea Consumption on Muscle Strength and Oxidative Stress Markers after Eccentric Exercise. *Br J Nutr* **2016**, *115*, 1370–1378, doi:10.1017/S000711451600043X.

5. Panza, V.P.; Brunetta, H.S.; de Oliveira, M.V.; Nunes, E.A.; da Silva, E.L. Effect of Mate Tea (Ilex Paraguariensis) on the Expression of the Leukocyte NADPH Oxidase Subunit P47phox and on Circulating Inflammatory Cytokines in Healthy Men: A Pilot Study. *International Journal of Food Sciences and Nutrition* **2019**, *70*, 212–221, doi:10.1080/09637486.2018.1486393.

6. Ruskovska, T.; Morand, C.; Bonetti, C.I.; Gebara, K.S.; Cardozo Junior, E.L.; Milenkovic, D. Multigenomic Modifications in Human Circulating Immune Cells in Response to Consumption of Polyphenol-Rich Extract of Yerba Mate (Ilex Paraguariensis A. St.-Hil.) Are Suggestive of Cardiometabolic Protective Effects. *Br J Nutr* **2023**, *129*, 185–205, doi:10.1017/S0007114522001027.

7. Bazyar, H.; Moradi, L.; Zaman, F.; Zare Javid, A. The Effects of Rutin Flavonoid Supplement on Glycemic Status, Lipid Profile, Atherogenic Index of Plasma, Brain-Derived Neurotrophic Factor (BDNF), Some Serum Inflammatory, and Oxidative Stress Factors in Patients with Type 2 Diabetes Mellitus: A Double-Blind, Placebo-Controlled Trial. *Phytother Res* **2023**, *37*, 271–284, doi:10.1002/ptr.7611.

8. Dehghani, F.; Sezavar Seyedi Jandaghi, S.H.; Janani, L.; Sarebanhassanabadi, M.; Emamat, H.; Vafa, M. Effects of Quercetin Supplementation on Inflammatory Factors and Quality of Life in Post-Myocardial Infarction Patients: A Double Blind, Placebo-Controlled, Randomized Clinical Trial. *Phytother Res* **2021**, *35*, 2085–2098, doi:10.1002/ptr.6955.

9. Bailly, A.R.; Hester, G.M.; Alesi, M.G.; Buresh, R.J.; Feito, Y.; Mermier, C.M.; Ducharme, J.B.; VanDusseldorp, T.A. Quercetins Efficacy on Bone and Inflammatory Markers, Body Composition, and Physical Function in Postmenopausal Women. *J Bone Miner Metab* **2025**, *43*, 304–314, doi:10.1007/s00774-025-01592-0.

10. Mury, P.; Dagher, O.; Fortier, A.; Diaz, A.; Lamarche, Y.; Noly, P.-E.; Ibrahim, M.; Pagé, P.; Demers, P.; Bouchard, D.; et al. Quercetin Reduces Vascular Senescence and Inflammation in Symptomatic Male but Not Female Coronary Artery Disease Patients. *Aging Cell* **2025**, *24*, e70108, doi:10.1111/acel.70108.

11. Brun, L.R.; Henríquez, M.M.; Stieben, L.A.R.; Cusumano, M.; Wilches-Visbal, J.H.; Saraví, F.D.; Brance, M.L. Positive Effect of Yerba Mate (Ilex Paraguariensis) Consumption on Bone Mineral Density in Postmenopausal Women Assessed by Dual Energy X-Ray Absorptiometry-Based 3-Dimensional Modeling. *J Bone Metab* **2025**, *32*, 123–132, doi:10.11005/jbm.24.827.

Supplementary Table 2. Risk of bias for in vitro studies. Risk of Bias assessment: OHAT tool for *in vitro* studies.

Study Reference	Design	D1	D2	D3	D4	D5	Overall RoB	Support for Judgement (Key Points)
Zhao et al., 2020 [1]	In vitro mechanistic	High	High	Some Concerns	Low	High	High	D1: High risk - Commercial RA-FLS cell line used; no mention of authentication testing (e.g., for mycoplasma) or randomization of cell plating/treatment allocation. D2: High risk - No description of blinding during treatment; culture conditions (CO_2, temperature) not specified. D3: Some Concerns - Transwell counting and immunofluorescence

								likely not blinded; methods are standard but lack validation details. D4: Low risk - All experiments repeated at least three times; no mention of data exclusion. D5: High risk - Western blot data for key targets like IRAK1/TRAF6 (known miR-146a targets) mentioned in discussion but not investigated or reported; potential selective reporting of favorable mechanistic pathways.
Chen et al., 2020 [2]	In vitro co-culture	Some Concerns	High	Some Concerns	Low	Some Concerns	Some Concerns	D1: Some Concerns - UCMSCs from 3 donors with full characterization (flow cytometry, differentiation), PBMCs from healthy volunteers; no randomization mentioned. D2: High risk - No blinding of treatment administration; culture conditions specified (37°C, 5% CO_2) but no monitoring details. D3: Some Concerns - Flow cytometry analysis likely standardized but blinding not mentioned; Western blot quantification methods not fully described. D4: Low risk - All experiments performed in triplicate (n=3); no data exclusions mentioned. D5: Some Concerns - All primary outcomes reported but IL-10 data mentioned as "not affected" without showing results; limited dose-response data for quercetin.

Baeza et al., 2016 [3]	In vitro antioxidant	Low	Some Concerns	Low	Low	Low	Low	D1: Low risk - HepG2 cell line well characterized; multiple concentrations tested; appropriate controls included. D2: Some Concerns - Standard culture conditions (37°C, 5% CO_2) used but no mention of blinding during treatment administration. D3: Low risk - Multiple validated assays used (LDH, ROS, GSH, enzyme activities, MDA, carbonyls); methods well described and standardized. D4: Low risk - All experiments performed with adequate replicates (n=4-12); complete data reporting. D5: Low risk - All measured outcomes reported; dose-response relationships clearly presented.

Sun et al., 2021 [4]	In vitro mechanistic	Some Concerns	Some Concerns	High	Low	High	High	D1: Some Concerns - Primary commercial RA-FLS cell line was used. While this source is reputable, the lack of a specific statement on cell authentication testing introduces uncertainty. D2: Some Concerns - Quercetin and TNF-α concentrations specified; but lack of details on compound purity/vehicle and culture conditions (CO2 not specified for most assays). D3: High risk - No mention of randomization of cell plating or treatment allocation across experimental groups. D4: Low risk - All experiments repeated three times; no mention of data exclusion. D5: High risk - No statement of blinding during outcome assessment (e.g., for ELISA, Western blot analysis, cell counting).
Chen et al., 2020 [5]	In vitro	High	High	Some Concerns	Low	Low	High	D1: No mention of randomization for cell culture treatment allocation or plate placement. D2: No statement on blinding of personnel during treatment administration. D3: Standard, validated methods were used, but no information on blinding during outcome assessment. D4: All experiments were performed in replicates (e.g., triplicate or quadruplicate) and data appear completely reported. D5: All outcomes described in the methods are fully reported in the results.
Wang et al., 2019 [6]	*In vitro*	High	High	Some Concerns	Low	Low	**High**	**D1:** No mention of randomization for cell culture treatment allocation. **D2:** No statement on blinding of personnel during treatment administration. **D3:** Objective methods were used, but no information on blinding during outcome assessment. **D4:** Complete outcome data reported for all replicates. **D5:** All measured outcomes specified in the methods are reported.
Santos et al., 2025 [7]	*In vitro* / *In silico*	High	High	Some Concerns	Low	Low	**High**	**D1:** No mention of randomization for cell culture treatment allocation or plate placement. **D2:** No statement on blinding of personnel during treatment administration. **D3:** Standard, validated methods and appropriate controls were used, but no information on blinding during outcome assessment. **D4:** All experiments were performed in triplicate and data appear completely reported (n-values, statistical analysis). **D5:** All outcomes described in the methods (viability, oxidative stress,

- **D1:** Selection Bias (Allocation/randomization of experimental units, e.g., cell seeding, plate placement)
- **D2:** Performance Bias (Standardization of experimental conditions, blinding of personnel during treatment)
- **D3:** Detection Bias (Blinding during outcome assessment, use of validated methods, appropriate controls)

- **D4:** Attrition/Exclusion Bias (Complete reporting of replicates, justification for exclusions)
- **D5:** Selective Reporting Bias (Reporting of all measured outcomes, availability of raw data)

Possible Judgements:

- *Low risk*
- *Some concerns*
- *High risk*

Bibliography

1. Zhao, J.; Chen, B.; Peng, X.; Wang, C.; Wang, K.; Han, F.; Xu, J. Quercetin Suppresses Migration and Invasion by Targeting miR-146a/GATA6 Axis in Fibroblast-like Synoviocytes of Rheumatoid Arthritis. *Immunopharmacol Immunotoxicol* **2020**, *42*, 221–227, doi:10.1080/08923973.2020.1742732.

2. Chen, G.; Ye, Y.; Cheng, M.; Tao, Y.; Zhang, K.; Huang, Q.; Deng, J.; Yao, D.; Lu, C.; Huang, Y. Quercetin Combined With Human Umbilical Cord Mesenchymal Stem Cells Regulated Tumour Necrosis Factor-α/Interferon-γ-Stimulated Peripheral Blood Mononuclear Cells via Activation of Toll-Like Receptor 3 Signalling. *Front Pharmacol* **2020**, *11*, 499, doi:10.3389/fphar.2020.00499.

3. Baeza, G.; Sarriá, B.; Mateos, R.; Bravo, L. Dihydrocaffeic Acid, a Major Microbial Metabolite of Chlorogenic Acids, Shows Similar Protective Effect than a Yerba Mate Phenolic Extract against Oxidative Stress in HepG2 Cells. *Food Res Int* **2016**, *87*, 25–33, doi:10.1016/j.foodres.2016.06.011.

4. Sun, H.-T.; Li, J.-P.; Qian, W.-Q.; Yin, M.-F.; Yin, H.; Huang, G.-C. Quercetin Suppresses Inflammatory Cytokine Production in Rheumatoid Arthritis Fibroblast-like Synoviocytes. *Exp Ther Med* **2021**, *22*, 1260, doi:10.3892/etm.2021.10695.

5. Chen, X.; Yang, J.H.; Cho, S.S.; Kim, J.H.; Xu, J.; Seo, K.; Ki, S.H. 5-Caffeoylquinic Acid Ameliorates Oxidative Stress-Mediated Cell Death via Nrf2 Activation in Hepatocytes. *Pharm Biol* **2020**, *58*, 999–1005, doi:10.1080/13880209.2020.1818791.

6. Wang, S.; Sarriá, B.; Mateos, R.; Goya, L.; Bravo-Clemente, L. TNF-α-Induced Oxidative Stress and Endothelial Dysfunction in EA.Hy926 Cells Is Prevented by Mate and Green Coffee Extracts, 5-Caffeoylquinic Acid and Its Microbial Metabolite, Dihydrocaffeic Acid. *International Journal of Food Sciences and Nutrition* **2019**, *70*, 267–284, doi:10.1080/09637486.2018.1505834.

7. Santos, J.M.D.; Touguinha, L.; Bridi, R.; Andreazza, A.C.; Bick, D.L.U.; Davidson, C.B.; Dos Santos, A.F.; Machado, K.A.; Scariot, F.J.; Delamare, L.A.P.; et al. Could the Inhibition of Systemic NLRP3 Inflammasome Mediate Central Redox Effects of Yerba Mate? An in Silico and Pre-Clinical Translational Approach. *J Ethnopharmacol* **2025**, *344*, 119518, doi:10.1016/j.jep.2025.119518.

Supplementary Table 3. Risk of bias for animal studies. Risk of Bias assessment: SYRCLE tool.

Study	D1	D2	D3	D4	D5	D6	D7	D8	D9	D10	Overall

de Carvalho et al., 2016 [1]	Unclear	Low	Unclear	Unclear	Unclear	Unclear	High	Low	Unclear	Unclear	High
Luz et al., 2016 [2]	Unclear	Low	Unclear	Unclear	Unclear	Unclear	High	Low	Unclear	Unclear	High
Pereira et al., 2017 [3]	Unclear	Low	Unclear	Unclear	Unclear	Unclear	High	Low	Unclear	Unclear	High
Correa et al., 2019 [4]	Unclear	Low	Unclear	Unclear	Unclear	Unclear	High	Low	Unclear	Unclear	High
Olate-Briones et al., 2024[5]	Unclear	Low	Unclear	Unclear	Unclear	Unclear	High	Low	Unclear	Unclear	High

**Study**	**Key points supporting risk-of-bias judgement**
**de Carvalho et al., 2016**	Randomization, allocation concealment, random housing, blinding of caregivers/assessors not reported. Manual nociceptive scoring without blinding. Complete data. Potential confounding from handling/stress.
**Luz et al., 2016**	No randomization or concealment described. Caregivers and assessors not blinded. Multiple subjective outcomes (cell counts, histology). Complete data. No preregistration; risk of selective reporting.
**Pereira et al., 2017**	No randomization/concealment. Caregivers not blinded. Only IHC clearly blinded; histomorphometry not. Complete data. Possible batch variability in extract; potential confounders (perimenopausal variability).
**Correa et al., 2019**	No randomization, concealment, or blinding. Several subjective assessments (paw volume, histology). Complete data. No preregistered protocol; multiple outcomes increase selective reporting risk.
**Olate-Briones et al., 2024**	No randomization/concealment. Caregivers unblinded. Outcome assessments largely unblinded except one. Mixed-sex groups may introduce variability. Complete data. Possible batch variability; no preregistration.

Bibliography

1. de Carvalho, E.F.; de Oliveira, S.K.; Nardi, V.K.; Gelinski, T.C.; Bortoluzzi, M.C.; Maraschin, M.; Nardi, G.M. Ilex Paraguariensis Promotes Orofacial Pain Relief After Formalin Injection: Involvement of Noradrenergic Pathway. *Pharmacognosy Res* **2016**, *8*, S31-37, doi:10.4103/0974-8490.178643.

2. Luz, A.B.G.; da Silva, C.H.B.; Nascimento, M.V.P.S.; de Campos Facchin, B.M.; Baratto, B.; Fröde, T.S.; Reginatto, F.H.; Dalmarco, E.M. The Anti-Inflammatory Effect of Ilex Paraguariensis A. St. Hil (Mate) in a Murine Model of Pleurisy. *Int Immunopharmacol* **2016**, *36*, 165–172, doi:10.1016/j.intimp.2016.04.027.

3. Pereira, C.S.; Stringhetta-Garcia, C.T.; da Silva Xavier, L.; Tirapeli, K.G.; Pereira, A.A.F.; Kayahara, G.M.; Tramarim, J.M.; Crivelini, M.M.; Padovani, K.S.; Leopoldino, A.M.; et al. Llex Paraguariensis Decreases Oxidative Stress in Bone and Mitigates the Damage in Rats during Perimenopause. *Exp Gerontol* **2017**, *98*, 148–152, doi:10.1016/j.exger.2017.07.006.

4. Correa, V.G.; Sá-Nakanishi, A.B. de; Gonçalves, G. de A.; Barros, L.; Ferreira, I.C.F.R.; Bracht, A.; Peralta, R.M. Yerba Mate Aqueous Extract Improves the Oxidative and Inflammatory States of Rats with Adjuvant-Induced Arthritis. *Food Funct.* **2019**, *10*, 5682–5696, doi:10.1039/C9FO00491B.

5. Olate-Briones, A.; Albornoz-Muñoz, S.; Rodríguez-Arriaza, F.; Rodríguez-Vergara, V.; Aguirre, J.M.; Liu, C.; Peña-Farfal, C.; Escobedo, N.; Herrada, A.A. Yerba Mate (Ilex Paraguariensis) Reduces Colitis Severity by Promoting Anti-Inflammatory Macrophage Polarization. *Nutrients* **2024**, *16*, 1616, doi:10.3390/nu16111616.
